# *Pten* loss in Lgr5^+^ hair follicle stem cells promotes SCC development

**DOI:** 10.7150/thno.35467

**Published:** 2019-10-22

**Authors:** Haiyan Chen, Xusheng Wang, Yu Chen, Jimin Han, Deqiang Kong, Meishu Zhu, Xiaobing Fu, Yaojiong Wu

**Affiliations:** 1 Tsinghua-Berkeley Shenzhen Institute (TBSI), Tsinghua University, Guangdong, China; 2 State Key Laboratory of Chemical Oncogenomics, and the Shenzhen Key Laboratory of Health Sciences and Technology, the Graduate School at Shenzhen, Tsinghua University, Shenzhen, Guangdong, China; 3 School of Life Sciences, Tsinghua University, Beijing, China; 4 School of Pharmaceutical Sciences (Shenzhen), Sun Yat-sen University, Guangzhou, China; 5 Shenzhen Second People's Hospital (The First Hospital Affiliated to Shenzhen University), Guangdong China.; 6 Wound Healing and Cell Biology Laboratory, Institute of Basic Medical Science, Chinese PLA General Hospital, Beijing, China; Stem Cell and Tissue Regeneration Laboratory, The First Affiliated Hospital, General Hospital of PLA, Beijing, China.

**Keywords:** *Pten*, β-catenin, TNF, hair follicles, SCC

## Abstract

Accumulating data support that tissue stem cells give rise to cancer cells. Hair follicle stem cells (HFSCs) undergo cyclic quiescence and activation and may sever as the origin of cutaneous squamous cell carcinoma (SCC). *Pten* is a tumor suppressor gene that is frequently mutated in hereditary cancer syndromes such as Cowden disease, which is featured with papillomatosis in cutaneous tissues and hyperkeratosis in the acral region of the skin. Additionally, mice with keratinocyte-specific *Pten* deficiency (*k5-Pten^-/-^* mice) show epidermal hyperplasia and spontaneous tumor formation. However, the impact of *Pten* mutation in HFSCs, such as in Lgr5^+^ HFSCs, on SCC formation is unclear.

**Methods**: We established experiments with wildtype and *Lgr5-CreER; Pten^flox/flox^* mice, and used DMBA/TPA two-stage skin carcinogenesis model to explore the effect of *Pten* loss in Lgr5^+^ HFSCs of 3 weeks old mice in skin carcinogenesis. *In vitro* experiments (cell culture and protein expression analysis) are employed to investigate molecular mechanisms involved.

**Results**: *Pten* loss in Lgr5^+^ HFSCs promoted SCC formation, which was attenuated in *TNF^-/-^* mice. Notably, β-catenin loss in Lgr5^+^ HFSCs decreased the formation of SCC. In addition, *Pten* loss in cultured epidermal stem cells upregulated the levels of both phospho-Akt and β-catenin.

**Conclusion**: *Pten* loss in Lgr5^+^ cells induced Akt/β-catenin signaling, and SCCs can subsequently be raised as progeny from these primed Lgr5^+^ stem cells.

## Introduction

Accumulating evidence indicates that certain cancers such as leukemia arise from somatic stem cells with gene mutations 1, 2*.* Lgr5 marks hair follicle stem cells (HFSCs) located in the lower bulge and the secondary hair germ of the telogen hair follicle (HF) 3. Lgr5^+^ cells substantially contribute to the cycling portion of anagen HFs and take part in the reepithelialization in skin wound healing 3, 4. In addition, HFSCs with misactivated *Hedgehog (Hh)* signaling recruited to the epidermis by wounding contribute to basal cell carcinoma (BCC)-like lesions 5, 6. Moreover, progeny of Lgr5^+^ HFSCs contribute to papillomavirus-induced SCC, the second most common skin cancer 7. *Pten* (phosphatase and tensin homolog deleted on chromosome ten), is a tumor suppressor gene that frequently mutated in hereditary cancer syndromes such as Cowden disease, which is featured with papillomatosis in cutaneous tissues and hyperkeratosis in the acral region of the skin 8, 9, and many other cancers 10. Additionally, mice with keratinocyte-specific *Pten* deficiency show epidermal hyperplasia and spontaneous tumor formation 11. However, the impact of *Pten* mutation in HFSCs, such as in Lgr5^+^ HFSCs, on cutaneous squamous cell carcinoma (SCC) formation is unclear. β-catenin has also shown to be involved in the development of SCC. SCC exhibit a preferential nuclear location of β-catenin, and inhibition of β-catenin signaling significantly attenuates the growth of SCC cells 12-14. However, whether the activity of β‐catenin signaling in HFSCs affecting SCC formation is unclear. In addition, it is also desired to uncover the interaction between Pten/Akt and β‐catenin signaling in SCC formation. Furthermore, previous studies show that *TNF*^-/-^ mice are resistant to DMBA/TPA-induced skin SCC 15, suggestive of the involvement of TNF in SCC development. In this study, we found that mice with *Pten* knock out in Lgr5^+^ HFSCs showed increased incidences of skin papilloma and SCC upon DMBA/TPA induction, while double loss of *Pten /CTNNB1*(β‐catenin gene) or *Pten/TNF* in Lgr5^+^ HFSCs greatly diminished the tumorigenesis. Thus our data indicate that *Pten* loss in HFSCs greatly promotes the formation of SCCs, and β‐catenin and TNF are critically involved.

## Methods

### Mice

C57BL/B6 mice (6-week-old, female) were purchased from Guangdong Medical Laboratory Animal Center, Guangzhou, China.* Lgr5-GFP-Cre-ERT2 (Lgr5-CreER)* mice were obtained from Jackson Laboratory (Stock No.: 008875). The mice were crossed with *Pten^flox/flox^* mice (a gift from Dr. Hong Wu at the University of California, Los Angeles) to obtain* Lgr5-CreER;Pten ^flox/flox^* mice, whose identify were verified (Figure S1A-B). *Lgr5-CreER; Pten^flox/flox^* mice were crossed with *β-catenin^flox/flox^ mice* (B6.129-Ctnnb1^tm2Kem^/KnwJ, provided by Dr. Zhenge Luo, Institute of Neuroscience, CAS) to obtain *Lgr5-CreER; Pten^flox/flox^; β-catenin^flox/flox^* mice. *TNF* knockout mice (TNFα-KO, B6.129S6-Tnf^tm1Gkl/J^) were obtained from Jackson Laboratory. *Lgr5-CreER; Pten^flox/flox^* mice were crossed with *TNF* knockout mice to obtain* Lgr5-CreER; Pten^flox/flox^*; *TNF KO* (knock out) mice. *Lgr5-CreER; Pten^flox/flox^* mice were crossed with *Rosa-mTmG* mice (Jackson Laboratory, Stock No.: 007576) to get* Lgr5-CreER; Pten^flox/flox^; Rosa-mTmG* mice. To knock out *Pten, β-catenin,* in Lgr5 cells,* Lgr5-CreER; Pten^flox/flox^* mice, *Lgr5-CreER; Pten^flox/flox^;β-catenin^flox/flox^* mice, *Lgr5-CreER; Pten^flox/flox^*;* TNF KO* (knock out) mice, *Lgr5-CreER; Pten^flox/flox^; Rosa-mTmG* mice aged 3 weeks received an intraperitoneal injection of 100 μL of tamoxifen (TAM, Sigma Aldrich) in corn oil at a concentration of 10 mg/mL for three times. In addition, we used littermate mice for control in all genetic mice model involved experiments. Mice were randomly divided into groups using a random-number table. The animals were maintained in a temperature-controlled environment (20 ± 1 °C) with free access to food and water. All procedures were performed with the approval of Animal Ethics Committee of Shenzhen Center for Disease Control and Prevention (CDC).

### Tumor induction in mice

Skin SCC in mice was induced as previously described 16, 17. Briefly, 25 μg DMBA (Sigma Aldrich) in 200 μL acetone was applied to the dorsal skin after shaving. After 2 weeks, TPA (10 nmol) in 200 μL was applied to the same area twice weekly for up to 30 weeks. Skin specimens were collected 4 weeks and 9 weeks after DMBA treatment, and when papilloma and SCC formed. The number of tumors per mouse was counted each week as palpable mass >1 mm in size. Tumor volume was also estimated and recorded periodically 16, 18, 19.

### Immunofluorescence (IF) staining

Freshly obtained skin samples from mice back with hair removal were fixed in 4% paraformaldehyde for 8 h. Then were taken off water in 10%, 20% and 30% sucrose gradient for 8 h and embedded in Tissue Freezing Medium (SAKURA Tissue-Tek® OCT Compound). Frozen tissue sections of the skin were incubated with different primary antibodies at 4 ℃ overnight, which were anti-p-Akt (Ser473, 1:200, GTX28932,GeneTex), anti-Ki67 (1:100, 20Raj1, eBioscience), and anti-p-β-Catenin (Ser552, 1:100, 5651S, Cell Signaling Technology), anti-Pten (1:100, 138G6,Cell Signaling Technology), anti-TNF (1:100, 1F3F3D4, eBioscience), anti-CD11b (1:50, 101201, Biolegend), anti-GFP (1:100, 598, MBL), anti-Keratin 14 (1:100, 906004, Biolegend), anti-MHC-II (1:100, 14-4-4S, eBioscience). Followed by detection with a TRITC or FITC-conjugated secondary antibody. Nuclei were stained with 4, 6-diamidino-2-phenylin-dole (DAPI). After mounting, samples were visualized under confocal microscope (FV1000; Olympus, Tokyo, Japan) 20.

### Western blotting

Freshly obtained skin samples from mice with hair removal were prepared in a lysis buffer containing 1% Triton X-100, 1% deoxycholic acid, 2 mM CaCl_2_ and protease inhibitors (10 μg/mL leupeptin, 10 μg/mL aprotinin, 1.8 mg/mL iodoacetamide and 1 mmol/L phenylmethyl sulfonyl fluoride) and quantified with a BCA protein assay kit (Pierce). Equal amounts of total protein were subjected to electrophoresis on 12% Bis-Tris gels, transblotted onto nitrocellulose membranes and probed with different primary antibodies: anti-Pten (1:1000, 138G6, Cell Signaling Technology), anti-p-Akt (Ser473, 1:1500, GTX28932, GeneTex), anti-p-β-catenin antibody (Ser552, 1:1000, 5651S, Cell Signaling Technology), anti-p-Gsk-3β (Ser9) antibody (1:1000, 5558S, Cell Signaling Technology), and anti-p-β-catenin antibody (Ser675, 1:1000, 4176S, Cell Signaling Technology), anti-Akt antibody (1:1000, GTX121937, GeneTex), anti-β-catenin antibody (1:1000, 8480, Cell Signaling Technology), respectively, followed by a peroxidase-conjugated secondary antibody (KPL). Immunoreactive bands were detected using ECL kit according to the manufacturer's instructions. Subsequent reprobing using anti-GAPDH was performed for internal loading control.

### Isolation and culture of epidermal stem cells

Neonatal mouse dorsal skin was harvested from *Pten^flox/flox^;Rose-mTmG* mice 1~3 days after birth. The tissue was cut into 2~3 mm^2^ pieces, washed 3 times in HBSS, and digested with 0.3% Dispase II (sigma) for 90 min at 37 °C. The epidermis was manually removed from the tissue. Epithelial stem cells were isolated based on their high adhesive property 21. Briefly, the epithelial layer was cut into slurry and treated with 0.2% collagenase I (Sigma) for 60 min at 37 °C with shaking and filtered through a 40 µm cell strainer. The cells were seeded in tissue culture dishes coated with 50 µg/mL collagen I (Sigma) and incubated in CnT-07 PCT Epidermal Keratinocyte Medium (CnT-07; CELLnTEC Advanced Cell Systems, Bern, Switzerland) with supplements provided by the manufacture for 60 min. The non-adherent cells were removed and the adherent cells were maintained. When reaching 80% confluence, the culture was passaged after digestion with accutase (Sigma) 4.

### Transfection

Epidermal stem cells were seeded on 6 wells culture dishes, and when cells reached 40% confluence, the medium was exchanged with 1 mL new medium containing 40 µL 1.0×10^10^ pfu GFP-Cre-adenovirus and incubated for 24 h. Then the medium was changed to growth medium, and cells were collected after incubation for 24, 48 and 72 h.

### Histological analysis

Freshly obtained skin samples from mice back with hair removal were fixed in 10% formalin or other fixatives for 12-24 h at room temperature. After dehydration, tissues were embedded in paraffin. Tissue sections were rehydrated with 100% ethanol, 95% ethanol, 75% ethanol, and deionized H_2_O, 3 min each. Then use hematoxylin to stain nuclear and eosin to stain cytoplasm and visualized with a Leica microscope. The mean width of HFs was measured, and 100 HFs per mice were measured per mouse.

### Statistical analysis

Results are expressed as mean±s.e.m. unless stated otherwise. Statistical comparisons between two groups were evaluated by Student's t-test. A probability (*P*) value <0.05 was considered to indicate statistical significance.

## Results

### *Pten* loss in Lgr5^+^ stem cells induces hair follicle hyperplasia

**Figure 1:**
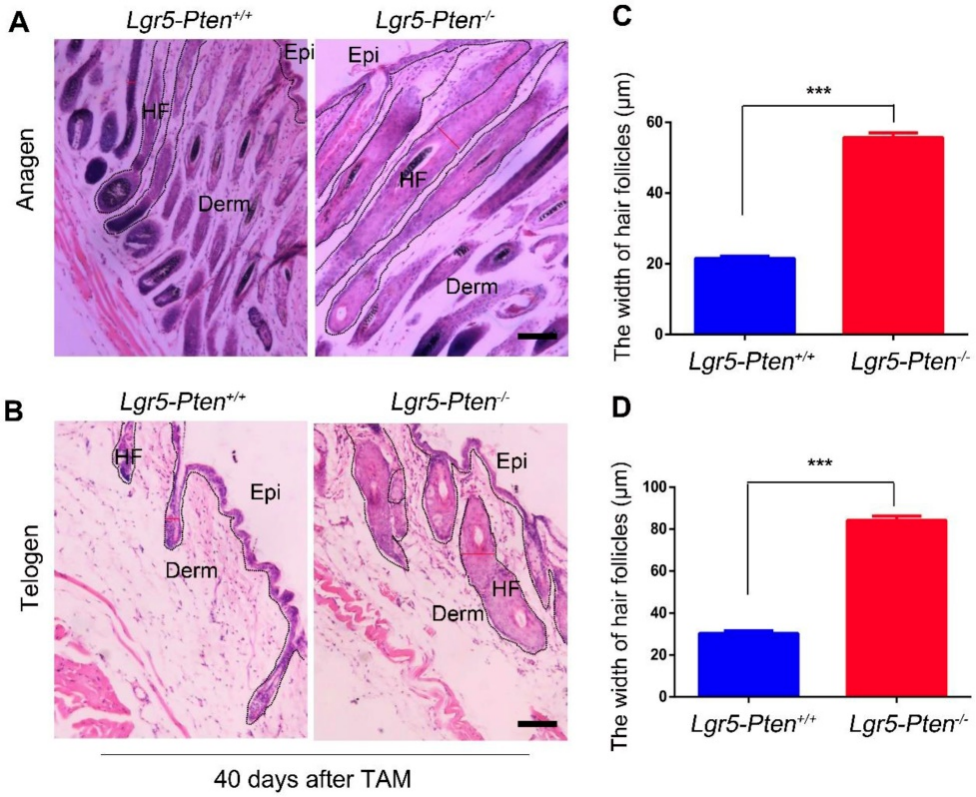
***Pten* loss in Lgr5^+^ HFSCs induces HF hyperplasia.** *Lgr5-CreER;Pten^flox/flox^* mice aged 3 weeks were treated with or without tamoxifen (TAM), and the skin tissues were harvested for histological analysis at different time points. **(A)** 20 days after TAM treatment, *Lgr5-Pten^-/-^*mice showed enlargement of the HF with increased cell number (hyperplasia). **(B)** 40 days after TAM treatment, in *Lgr5-Pten^-/-^*mice the HF entered telogen phase, but they were still larger than telogen HFs in *Lgr5-Pten^+/+^* mice.100 hair follicles per mouse were measured, 10 mice were analyzed. Scale bars, 100 μm. Epi, Epidermis; Derm, Dermis; HF, hair follicle. Data are expressed as the mean±s.e.m. ***P< 0.005.

A previous study showed that *Pten* deletion in Lgr5^+^ HFSCs in 7~8 weeks old mice did not induce the hyperproliferation of HFSCs 22. Here we examined the influence of *Pten* loss on Lgr5^+^ HFSCs in 3 weeks old *Lgr5-CreER;Pten^flox/flox^* mice. The mice were treated with tamoxifen to induce *Pten* loss in Lgr5^+^ cells (*Lgr5-Pten^-/-^*). At the 12^th^ day after intraperitoneal injection of tamoxifen, immunofluorescence staining of the skin tissue showed that Pten level was extensively reduced in Lgr5^+^ HFSCs, resulting in increased levels of p-Akt (Figure S2A-B). Notably, 20 days after tamoxifen administration, the mice showed significantly enlarged HF containing more cells (hyperplasia), compared to *Lgr5-CreER;Pten^flox/flox^* mice without tamoxifen treatment (*Lgr5-Pten^+/+^*) (Figure 1A, C). The enlarged HF shortened when proceeding into the telogen phase but still much wider than in the *Lgr5-Pten^+/+^* mice, as examined 40 days (8-9 weeks of age) after *Pten* deletion (Figure 1B, D). Taken together, the data indicate that *Pten* loss in Lgr5^+^ HFSCs induce HF hyperplasia in 3 weeks old mice.

### *Pten* loss in Lgr5^+^ stem cells promotes papilloma formation

**Figure 2:**
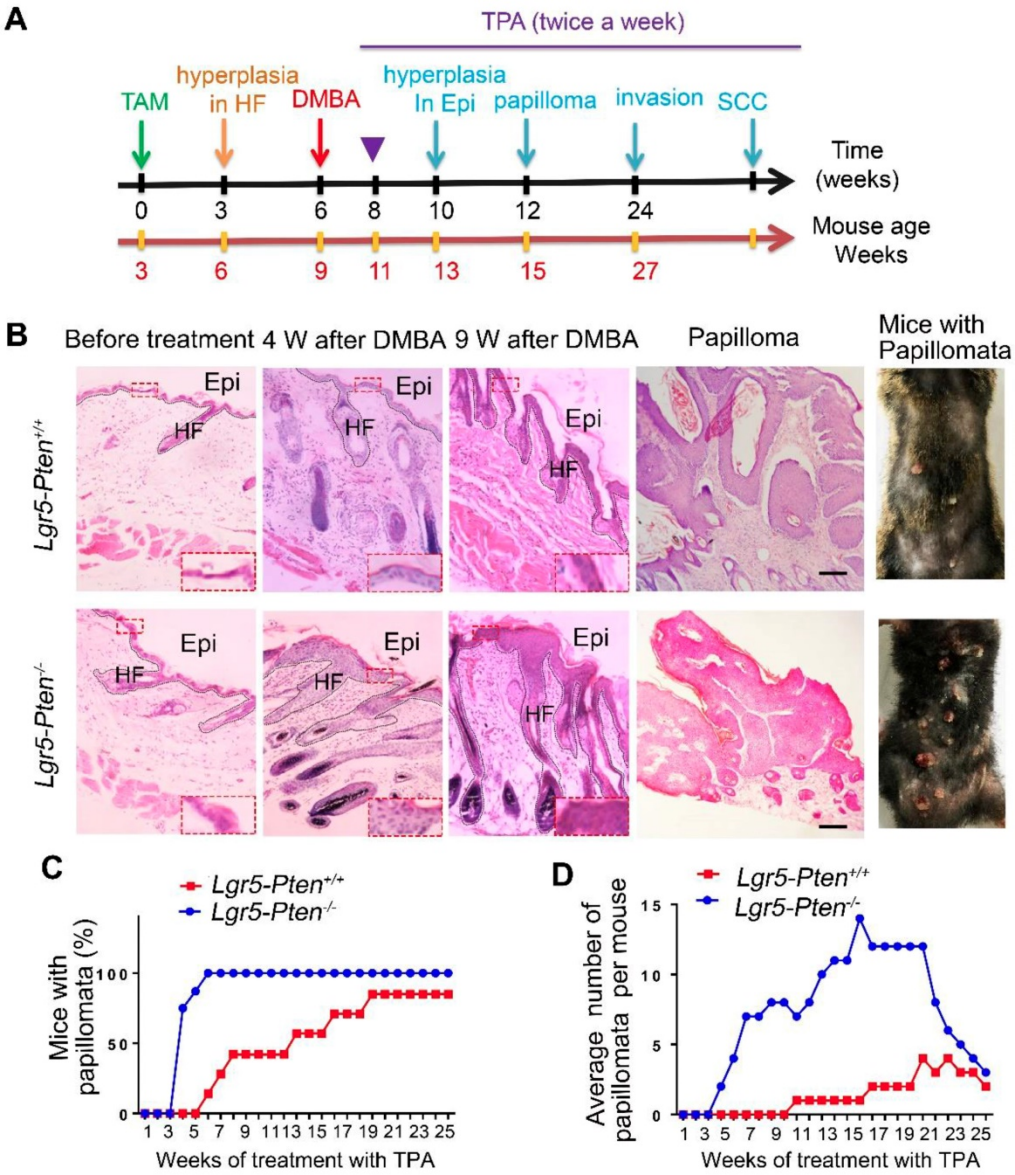
***Pten* loss in Lgr5^+^ HFSCs promotes papilloma formation. (A)** A schematic diagram of the two-step DMBA/TPA carcinogenesis assay. 3 weeks old *Lgr5-CreER;Pten^flox/flox^* mice were treated with (*Lgr5-Pten^-/-^*, n=8) or without (*Lgr5-Pten^+/+^*, n=7) tamoxifen (TAM), followed by treatments with DMBA/TPA. **(B)** Skin tissues were harvested at different time points for histological analysis. HE staining of tissue sections showed changes of the skin. **(C)** The incidence of mice with skin papillomata. **(D)** The average number of papillomata per mouse in of the two groups. Scale bars, 100 μm. W, weeks.

To gain insight into the role of *Pten* loss in Lgr5^+^ HFSCs in the development of SCCs, we employed DMBA/TPA SCC mouse model 16, 17. Six weeks after *Pten* deletion in *Lgr5-Pten^-/-^* mice, DMBA was applied to the dorsal skin of mice after hair shaving, followed by TPA treatment (Figure 2A). *Lgr5-Pten^+/+^* received equal treatment of DMBA/TPA were used as control. Four weeks after the DMBA treatment, the dorsal skin of mice showed hyperplasia in both *Lgr5-Pten^-/-^* mice and *Lgr5-Pten^+/+^* mice, whereas the epidermal layer of the skin was much thicker in *Lgr5-Pten^-/-^* mice (Figure 2B). Nine weeks after DMBA treatment, significant hyperplasia was observed in both groups, but the lesion was more severe in *Lgr5-Pten^-/-^* mice (Figure 2B). Importantly, skin papillomata were observed in* Lgr5-Pten^-/-^* mice early in the 5^th^ week after DMBA treatment, whereas in *Lgr5-Pten^+/+^* mice papillomata were found 8 weeks after DMBA treatment. In addition, the incidence of mice developing papillomata was higher in *Lgr5-Pten^-/-^* mice (100%, n=8) than in *Lgr5-Pten^+/+^* mice (85.7%, n=7) 25 weeks after TPA treatment (Figure 2C, D). Together, these data indicate that *Pten* knockout in Lgr5^+^ HFSCs greatly promotes skin papilloma formation.

### *Pten* loss in Lgr5^+^ HFSCs promotes SCC development

**Figure 3:**
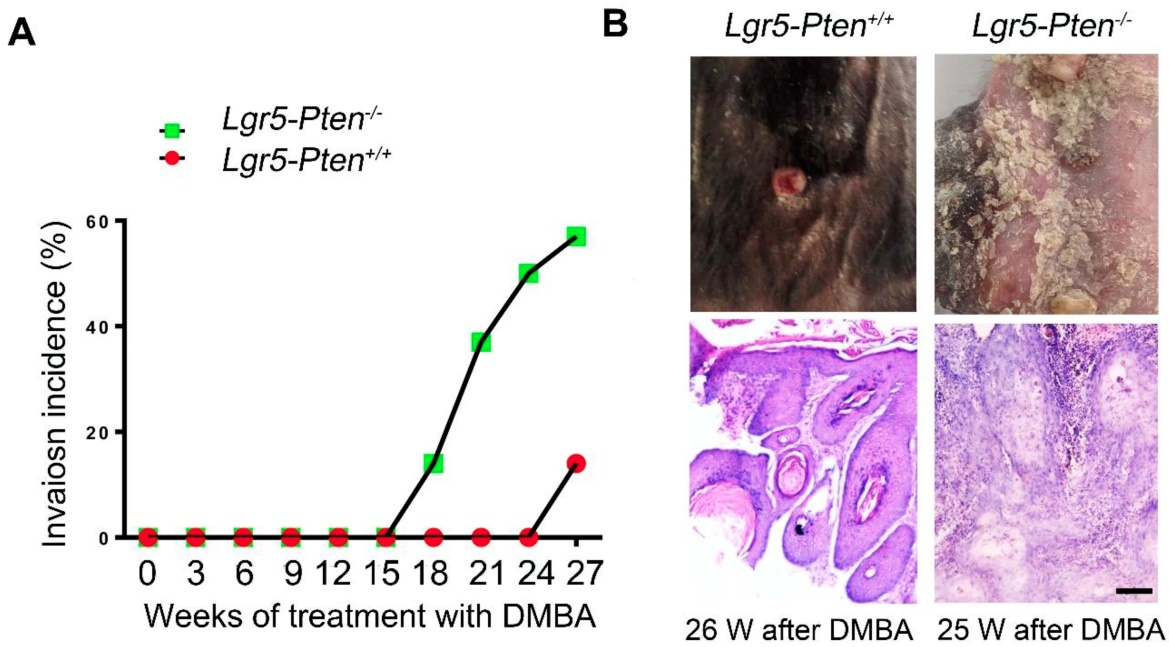
***Pten* loss in Lgr5^+^ HFSCs promotes SCC development. (A)** *Lgr5-Pten^-/-^* mice and *Lgr5-Pten^+/+^* mice were subjected to DMBA/TPA treatment, and the incidence of SCC in mice were examined. **(B)** Tumor tissues were harvested at different time points and subjected to histological analysis. HE staining of tissue sections showed invasion lesions. *Lgr5-Pten^-/-^* mice, n=8; *Lgr5-Pten^+/+^*mice, n=7. Scale bars, 100 μm.

We next examined whether *Pten* knockout in Lgr5^+^ HFSCs promoted skin SCC development. We found that some papillomata in* Lgr5-Pten^-/-^* mice developed into invasive lesions 18 weeks after DMBA treatment, where proliferating cells in the epidermis- and follicle-like structures invaded into the space between them in histology, exhibiting the feature of SCC; by contrast, similar lesions were found in much later in *Lgr5-Pten^+/+^* mice (26 weeks after DMBA treatment) (Figure 3A-B). 26 weeks after DMBA treatment, 62.5% of *Lgr5-Pten^-/-^* mice developed SCC lesions compared to 14.3% in *Lgr5-Pten^+/+^* mice (Figure 3 A). These data indicate that *Pten* deletion in Lgr5^+^ HFSCs contributes to the development of SCC.

### Lineage tracing of Lgr5^+^ HFSCs and their progeny in tumor development

**Figure 4:**
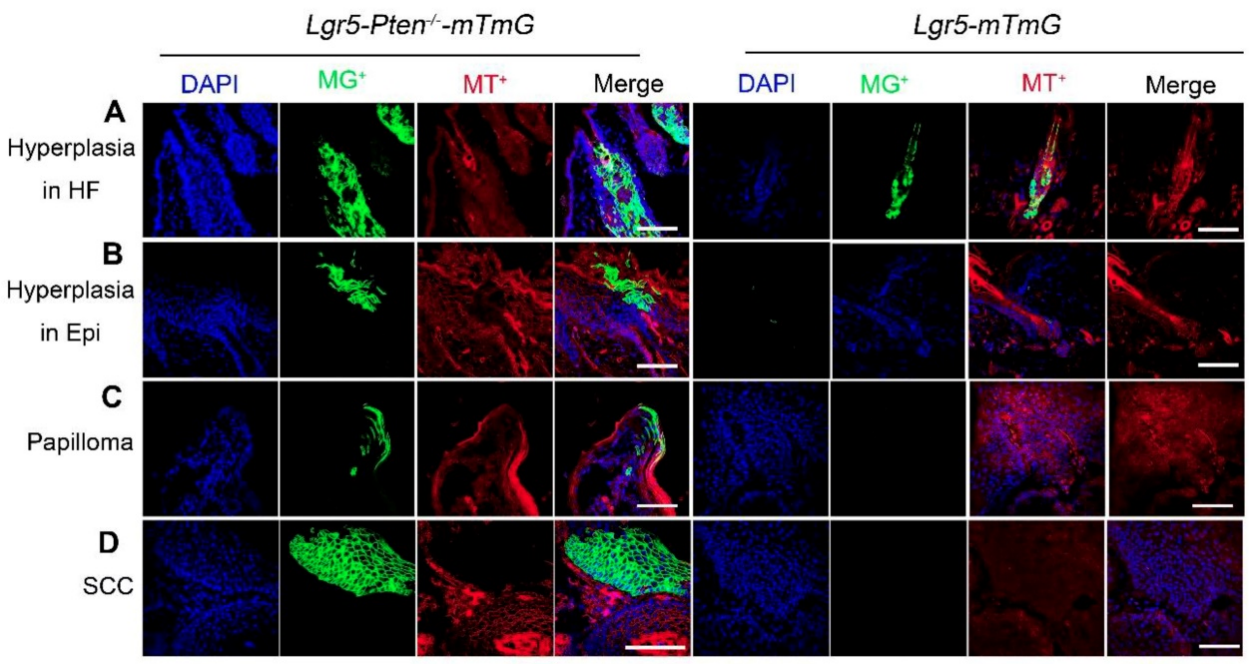
** Lineage tracing of Lgr5^+^ HFSCs and their progeny in papilloma and SCC development.** *Lgr5-Pten^-/-^-mTmG* mice (n=8) and *Lgr5-mTmG* mice (n=7) received treatment of DMBA/TPA. *Lgr5-Pten^-/-^-mTmG* mice developed HF and epidermal hyperplasia, which contained increased amount of mT^-^/mG^+^ cells derived from Lgr5^+^ HFSCs. With progression of the disease, mT^-^/mG^+^ cells were found in papillomata and in SCC in *Lgr5-Pten^-/-^-mTmG* mice, but not in *Lgr5-mTmG* mice. SCC, squamous cell carcinoma. Scale bars, 100 μm.

To further examine the contribution of Lgr5^+^ HFSCs with *Pten* loss and their progeny to skin tumor development, we used *Lgr5-CreER; Pten^flox/flox^; Rosa-mTmG* (*Lgr5-Pten^-/-^-mTmG*) and* Lgr5-CreER;Rosa-mTmG* (*Lgr5-mTmG*) mice (control). The mice were treated with DMBA/TPA as indicated in Figure 2A. The result showed that an increased amount of cells were progenies of Lgr5^+^ stem cells (membrane tdTomato^-^/membrane GFP^+^, mT^-^/mG^+^) in hyperplastic HFs and epidermis in *Lgr5-Pten^-/-^-mTmG* mice, whereas in *Lgr5-mTmG* mice the HF and epidermis did not show hyperplasia and contained fewer mT^-^/mG^+^ cells (Figure 4A, B). With progression of the disease, we found mT^-^/mG^+^ cells in papillomata and in SCC, which formed colonies, in *Lgr5-Pten^-/-^-mTmG* mice, but not in *Lgr5-mTmG* mice (Figure 4C, D). To verify the contribution of Lgr5^+^ cells (with *Pten* loss) in papilloma and SCC, we performed IF staining of tissue sections of these tumors derived from *Lgr5-Pten^-/-^* mice and *Lgr5-Pten^+/+^* mice for the expression of Lgr5, and we did not detect the presence of Lgr5-expressing cells (Figure S3). These data suggest that the progeny of Lgr5^+^ HFSCs with* Pten* loss contribute to skin papilloma and SCC.

### β-catenin effects at the downstream of Pten/Akt signaling

**Figure 5:**
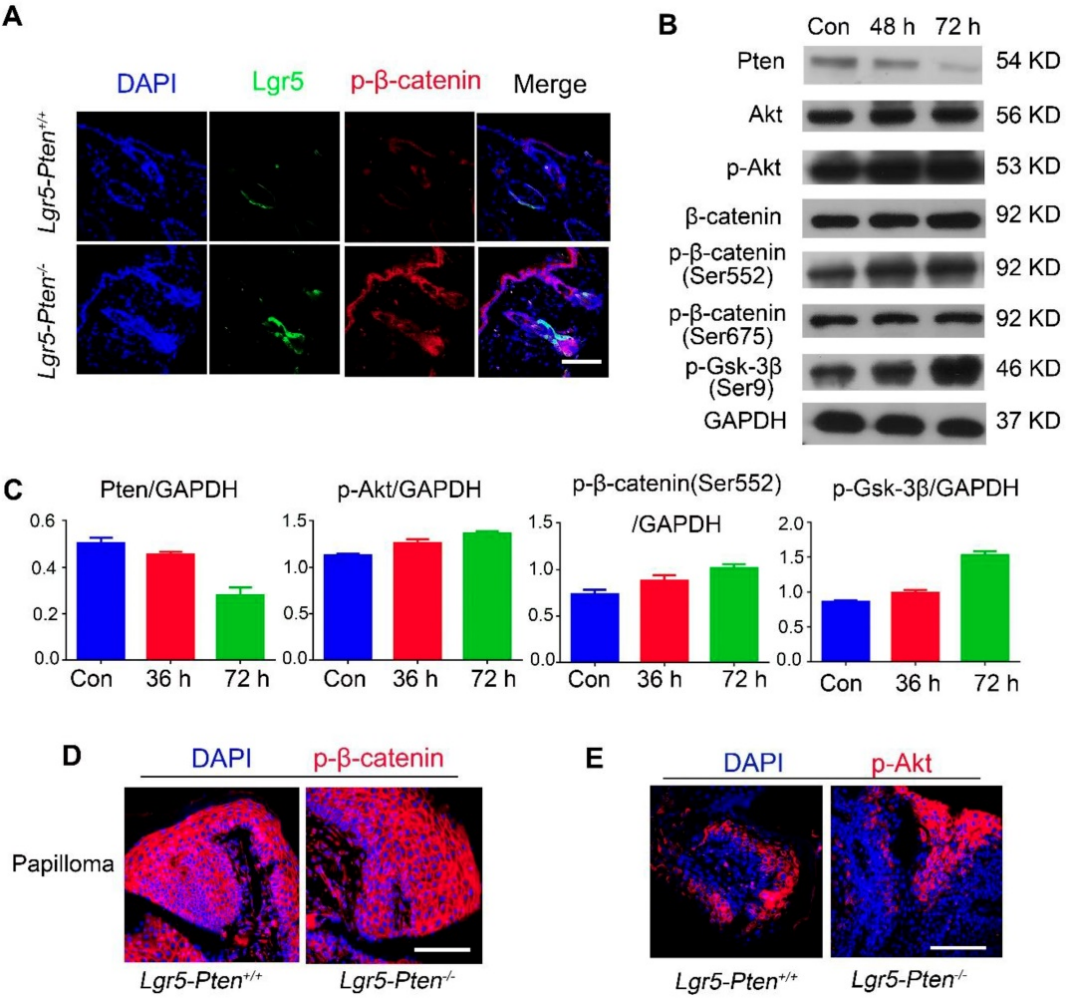
** β-catenin activity after* Pten* loss in epidermal stem cells and papillomata. (A)** 15 days after injection of tamoxifen for three times, marked p-β-catenin (Ser552) was detected in Lgr5^+^ HFSCs in *Lgr5-Pten^-/-^*mice. **(B)** Western blot analysis showed that Pten expression decreased in cultured epidermal stem cells derived from* Pten^flox/flox^;Rose-mTmG* mice, 48 and 72 h after infection with Ad-Cre viruses. There were no obvious changes in total Akt, total β-catenin, and p-β-catenin (Ser675), but significantly increased levels of p-β-catenin (Ser552), p-Akt, p-Gsk-3β (Ser9). Data were representative of 3~5 independent experiments. **(C)** The bands were subjected to densitometry analysis and normalized to GAPDH, *P <0.05; **P < 0.01. **(D, E)** Representative images of immunofluorescence analysis showed high levels of p-β-catenin (Ser552) and p-Akt in papillomata in *Lgr5-Pten^-/-^* mice and *Lgr5-Pten^+/+^*mice. Scale bars, 100 μm.

**Figure 6:**
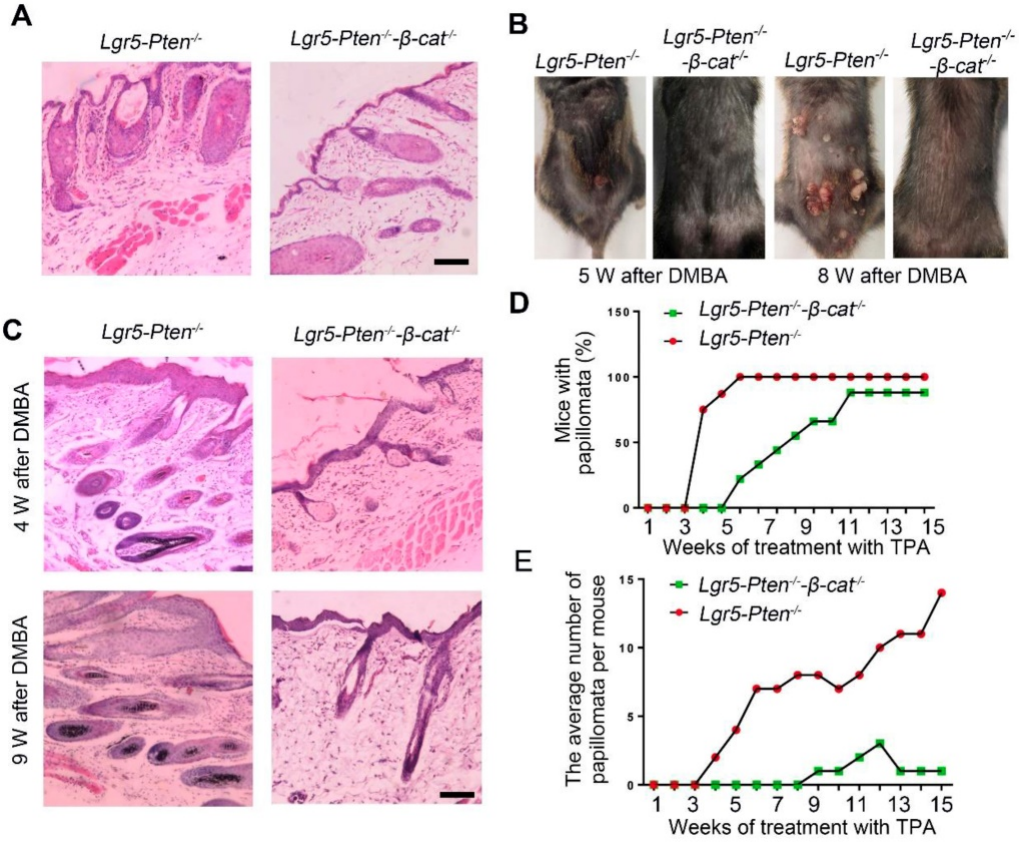
** The effect of β-catenin in *Pten* loss induced tumor formation. (A)** 40 days after injection tamoxifen for three times, histological analysis (HE staining) of the dorsal skin (in telogen phase) showed less severe HF hyperplasia in *Lgr5-Pten^-/-^-β-catenin^-/-^* mice compared to *Lgr5-Pten^-/-^* mice. **(B)** *Lgr5-Pten^-/-^-β-catenin^-/-^*mice developed fewer skin papillomata than* Lgr5-Pten^-/-^*mice. Images were taken 5 weeks and 8 weeks after DMBA/TPA treatment. **(C)** Histological analysis of the dorsal skins of *Lgr5-Pten^-/-^* mice and* Lgr5-Pten^-/-^-β-catenin^-/-^* mice 4 and 9 weeks after DMBA/TPA treatment, respectively. **(D)** The incidence of papilloma formation in *Lgr5-Pten^-/-^* mice and *Lgr5-Pten^-/-^-β-catenin^-/-^* mice. **(E)** The average number of papillomata in *Lgr5-Pten^-/-^*mice (n=8) and *Lgr5-Pten^-/-^-β-catenin^-/-^*mice (n=9). Scale bars, 100 μm. W, weeks.

To elucidate the molecular mechanisms underlying the role of *Pten* loss in Lgr5^+^ HFSCs in SCC development, we performed *in vitro* and *in vivo* experiments. Immunostaining of the skin tissue of *Lgr5-Pten^-/-^* mice showed elevated expression levels of p-β-catenin (Ser552) in the HF and epithelial cells (Figure 5A), indicating increased β-catenin signaling after *Pten* loss. To gain more insight into the crosstalk between Pten/Akt and β-catenin in epidermal stem cells (Epi-SCs), Epi-SCs were isolated from *Pten^flox/flox^;Rose-mTmG* mice and the cells were treated with Ad-Cre virus (Figure S4) to induce the knockout of *Pten*. Western blot showed that the expression of Pten decreased markedly at the 48 h and 72 h after Ad-Cre virus treatment, meanwhile the level of p-Akt increased, but the level of total Akt unchanged. In addition, increased levels of p-Gsk-3β (Ser9) and p-β-catenin (Ser552), but unchanged levels of p-β-catenin (Ser675) were also detected (Figure 5B, C). Consistently, elevated level of p-Akt and p-β-catenin (Ser552) were detected in papillomata (Figure 5D, E). Taken together, these data suggest *Pten* loss activates β-catenin possibly through the Akt mediated phosphorylation of β-catenin (Ser552). We detected more Ki67-expressing cells in papillomata of mice with *Pten* loss in Lgr5^+^ cells (Figure S5). To verify the role of β-catenin in *Pten* loss induced SCC formation, we generated *Lgr5-CreER;Pten^flox/flox^;β-catenin^flox/flox^*mice, whose *Pten* and *β-catenin* were deficient in Lgr5^+^ cells upon induction with tamoxifen (*Lgr5-Pten^-/-^-β-catenin^-/-^* mice); we found that less severe HF and epidermal hyperplasia in the mice compared to *Lgr5-Pten^-/-^* mice (Figure 6A, C). Moreover, the incidence of tumor formation and the average number of papillomata per mouse also decreased markedly in *Lgr5-Pten^-/-^-β-catenin^-/-^*mice (Figure 6B, D, E). These data suggest that the loss of *Pten* in Lgr5^+^ HFSCs is likely through β-catenin signaling to promote SCC development.

### TNF plays a crucial role in *Pten* loss induced tumor formation

**Figure 7:**
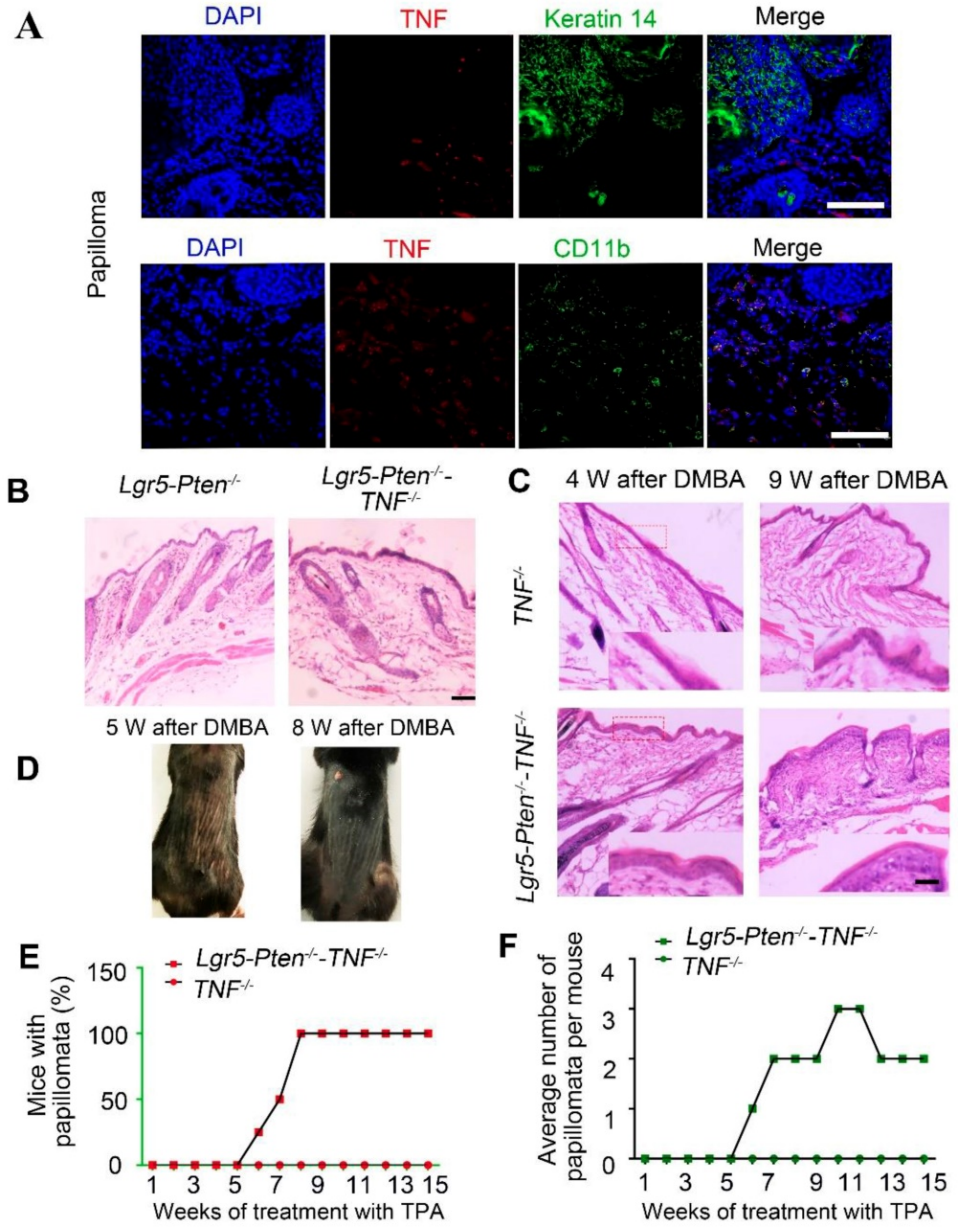
** TNF is necessary for *Pten* loss induce tumor formation. (A)** TNF expression was detected in papillomata and was largely present in CD11b^+^ cells.** (B)** 40 days after injection tamoxifen for three times, histological analysis (HE staining) of the dorsal skin of *Lgr5-Pten^-/-^*mice and* Lgr5-Pten^-/-^-TNF^-/-^*mice showed HF hyperplasia (telogen phase). **(C)** 4 and 9 weeks after DMBA/TPA treatment, epidermal hyperplasia in the dorsal skin of *Lgr5-Pten^-/-^-TNF^-/-^* mice was more obvious than in *TNF^-/-^*mice. **(D)** Representative images showing papillomata in* Lgr5-Pten^-/-^-TNF^-/-^* mice 5 weeks and 8 weeks after DMBA/TPA treatment. **(E)** The incidence of skin tumor development in* TNF^-/-^* mice and in *Lgr5-Pten^-/-^-TNF^-/-^* mice. (E) The average number of papillomata per mouse in* TNF^-/-^* mice (n=8) and in* Lgr5-Pten^-/-^-TNF^-/-^*mice (n=8). Scale bars, 100 μm. W, weeks.

TNF is a potent proinflammatory cytokine 23, 24. Previous studies have provided evidence that TNF is required for carcinogenesis 15, 25. We found that TNF was abundantly expressed in skin papillomata in immunostaining (Figure 7A), largely in the cells in the stroma, co-localizing to CD11b^+^ (Figure 7A) and MHC-II^+^ (Figure S6) expressing cells, probably macrophages. To evaluate the role of the TNF in Lgr5^+^ stem cells with *Pten* loss induced SCC development, *Lgr5-CreER;Pten^flox/flox^;TNFa^-/-^* mice were generated and subjected to skin tumor induction using the DMBA/TPA protocol. The results showed that less severe HF hyperplasia in* Lgr5-CreER;Pten^flox/flox^;TNFa^-/-^* mice treated with tamoxifen (*Lgr5-Pten^-/-^-TNF^-/-^*) compared to *Lgr5-Pten^-/-^* mice (Figure 7B). After DMBA/TPA treatment, *Lgr5-Pten^-/-^-TNF^-/-^*mice displayed more severe epidermal hyperplasia compared to *TNF^-/-^* mice (Figure 7C). Consistently, reduced incidence of papillomata was found in *Lgr5-Pten^-/-^-TNF^-/-^* mice than in* Lgr5-Pten^-/-^* mice (Figure 2C, Figure 7D-E). Moreover, the average number of papillomata per mouse in *Lgr5-Pten^-/-^-TNF^-/-^*mice was much lower compared to that in *Lgr5-Pten^-/-^* mice (Figure 2D, Figure 7F), and *TNF^-/-^* mice did not develop papilloma when receiving the same DMBA /TPA treatment (Figure 7E-F). These data indicate that TNF has a crucial role in Lgr5^+^ HFSCs with* Pten* loss induced papilloma formation.

## Discussion

Lgr5-expressing cells have been found in many tumors, such as colon cancer 26, 27, papillary thyroid cancer 28, breast cancer 29, and gastric cancer 30. Recently, Lgr5 has been found to induce epithelial-mesenchymal transition (EMT) in human hepatocellular carcinoma cells and inhibit their apoptosis, resulting in drug resistance 31. In human skin SCC, high levels of Lgr5 have been found in immunohistological analysis 32. In the skin of mice, Lgr5^+^ stem cells in the HF undergo active cyclic proliferation, and contribute to epidermal cells after wounding to the skin 3, 4; but lineage tracing of Lgr5^+^ cells did not show their direct contribution to skin SCC, nor their progeny 33, 34. White and colleagues found that gain of oncogene *Ras* or the loss of tumor suppressors *p53* or* Pten* in HFSCs are unable to initiate tumors during the telogen phase of the HF in adult mice, suggesting that the mechanisms that keep HFSCs quiescent are dominant over the oncogenic influences. However, gain of* Kras^G12D^*, accompanied with loss of both *Pten* and *p53* was sufficient to induce skin malignancies in quiescent HFSCs. These data support that *Pten* has a crucial role in maintaining quiescence in the presence of tumorigenic stimuli 22. While it would be interesting to explore the role of *Pten* loss in skin malignancy development without other tumorigenic stimuli, *Pten-*only deletion in Keratin 15^+^ cells during HFSC quiescence did not result in hyperplasia in adult mice 28. However, in this study, we found that *Pten* deletion in Lgr5^+^ HFSCs at the telogen/anagen transition of 3 weeks old mice induced hyperplasia in HFs, and the mice showed more severe hyperplasia in the epidermis, larger number of papillomata and increased incidence of SCC. These data support that *Pten* deletion alone in the activated HFSCs is sufficient to drive these stem cell into hyperplasia. Our data further support that *Pten* loss induced SCC development is dependent on the activation of HFSCs. Lgr5^+^ HFSCs are the first activated stem cell population in response to anagen-initiating signals, whereas keratin 15^+^ HFSCs remain quiescent even in anagen phase 3. This may explain, at least in part, why *Pten* deletion alone in Lgr5^+^ HFSCs is sufficient to drive HFs to hyperplasia, whereas a combination of *Kras^G12D^* and the loss of both* Pten* and *p53* are needed to derive the Keratin 15^+^ HFSCs into hyperplasia. However, more concrete studies should be performed to deeply uncover the differential response of stem cells in activation and in quiescence to tumorigenic stimuli.

Wnt/β-catenin exhibits complicated effect on both the development of stem cells and cancers 35-38. In the HF, the transit-amplifying matrix compartment appears to be the target for malignant transformation by mutational activation of the Wnt cascade 39. Constitutive activation of β-catenin in HFs induces pilomatricoma-like lesions, where the exterior zone of the tumor is composed of densely packed cells resembling the matrix of HFs 40. Another HF tumor, namely trichofolliculoma, was developed in tamoxifen-inducible β-catenin transgene mice 41. Notably, the most spontaneous pilomatricomas in humans are featured with activating mutations in β-catenin 42. Recent evidence has shown that in many organs, such as the HF, resident adult stem cells can be the initiating cells for cancers 43, 44. In this study, deletion of both *Pten* and β-catenin in Lgr5^+^ HFSCs result in reduced HF hyperplasia and SCC development. Together with the *in vitro* data, our study support that the effect of β-catenin is downstream of Pten/Akt signaling in the initiation and development of SCC. However, more studies with gain and lose function of β-catenin and Pten/Akt signaling in HFSCs are desired, to further explore the crosstalk between Pten/Akt and β-catenin and its role in skin cancer development. In addition, our data suggest that TNF plays an essential role in *Pten* loss in HFSCs induced hyperplasia and SCC development. However, the relationship between TNF and Pten/Akt signaling in HFSCs activation and SCC development was still largely unknown. Given the evidence of TNF as a key component of HFSCs activation 4, 45, TNF and Pten/Akt signaling pathways possibly exhibit synergistic effect on SCC development via activating and transforming HFSC, respectively.

Conclusively, our study provide evidence that *Pten* loss in activating Lgr5^+^ HFSCs was sufficient to drive HFs into hyperplasia, and the *Pten* loss in Lgr5^+^ HFSCs plays an important role in subsequent SCC formation. Moreover, we found both β-catenin and TNF play essential roles in HFSCs with *Pten* loss induced hyperplasia and SCC development. Together, these data indicate that *Pten* loss in HFSCs is a key driver in SCC initiation and development, where active interaction of Pten/Akt signaling with other signals such as β-catenin and TNF are required.

## Supplementary Material

Supplementary figures.
