# Supplementary material for: Pten loss in Lgr5+ hair follicle stem cells promotes SCC development
Source: Theranostics. 2019 Oct 22;9(26):8321–31. doi: 10.7150/thno.35467 (PMC6857063; doi:10.7150/thno.35467)
Supplement: Supplementary file 1 — Supplementary figures. [file thnov09p8321s1.pdf]

Supplementary Figures

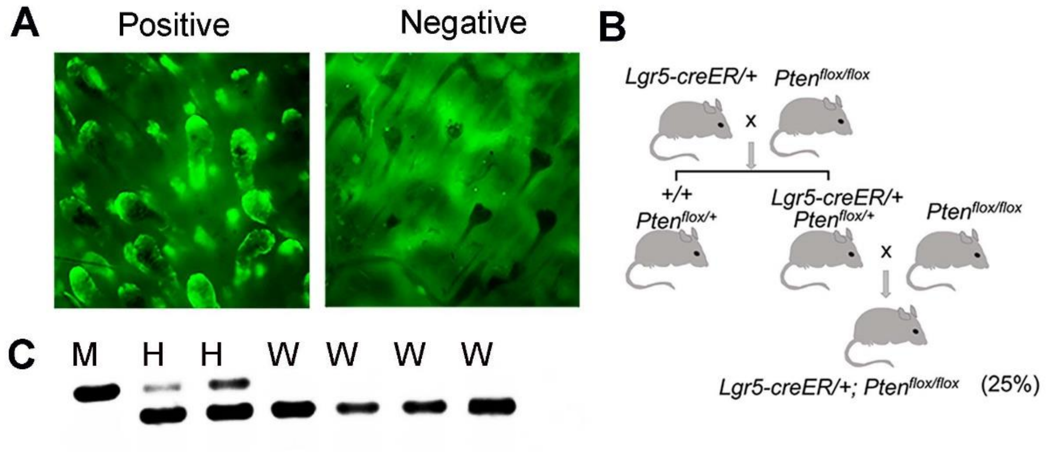

Figure S1. (A) *Lgr5-EGFP-IRES-creERT2* positive and negative hair follicles. **(B)** The mating map of *Lgr5-CreER;Pten*$^{flox/flox}$ mice. The genotype of *Pten*$^{flox/flox}$ mice. M: mutant; H: heterozygote; W: wild type.

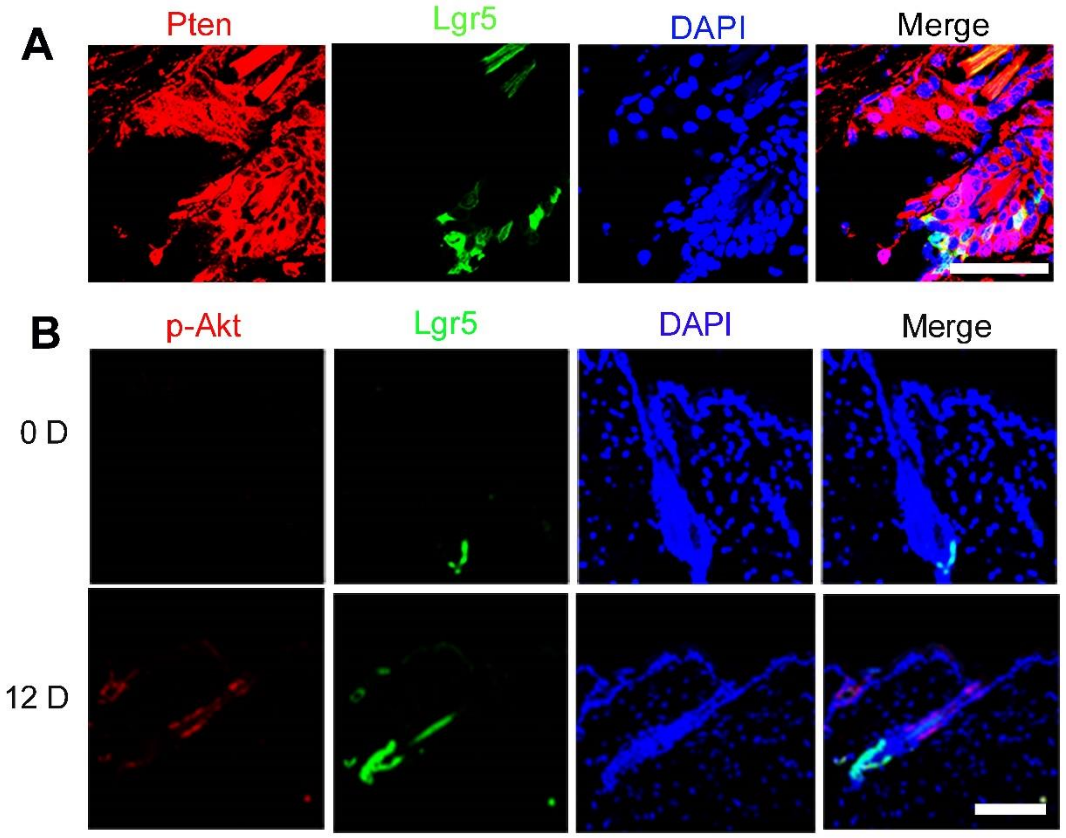

Figure S2. After intraperitoneal injection of tamoxifen for 12 days in *Lgr5-CreER; Pten*$^{flox/flox}$ mice, immunofluorescence stain analysis of the dorsal skin tissue of the mice did not detect the expression of Pten in Lgr5^{+} HFSCs (A), while the expression of p-Akt in Lgr5^{+} HFSCs increased (B). Scale bars, 100 μm. D, days.

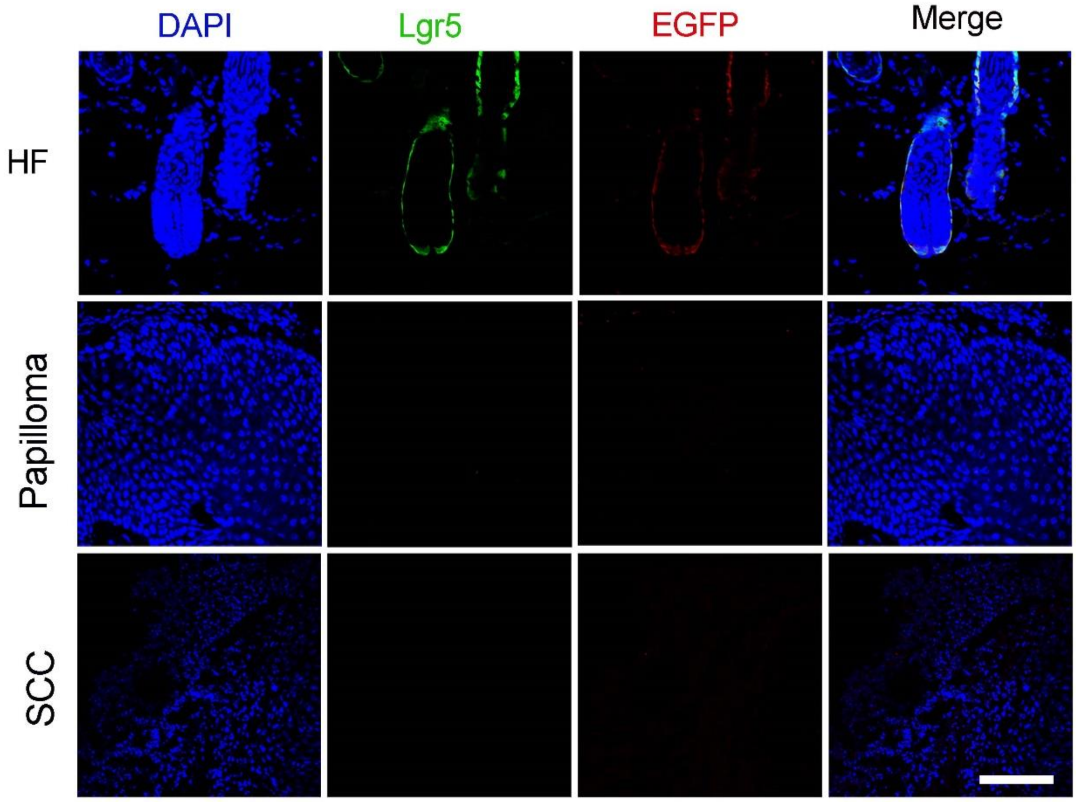

Figure S3. Expression of Lgr5 in papilloma and SCC. Tissue sections of the dorsal skin of *Lgr5-CreER;Pten*$^{flox/flox}$ mice, and papillomata and SCCs form *Lgr5-Pten*$^{-/-}$ mice were subjected to immunofluorescence analysis for Lgr5 expression. Lgr5-expressing cells were detected in the HF of *Lgr5-CreER;Pten*$^{flox/flox}$ mice which were also positive for EGFP, but not in papillomata and SCCs. Scale bar, 100 μm.

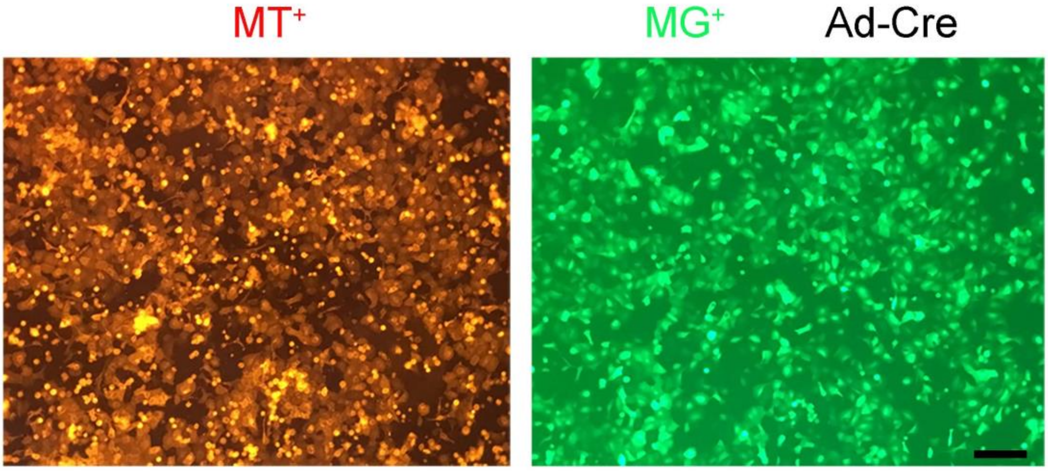

Figure S4. Epidermal stem cells isolated from *Pten*$^{flox/flox}$*; Rosa-mTmG* mice were cultured and infected with 40 μL 1×10^{10} pfu Ad-Cre virus. After 24 h, cells were viewed under florescence microscope. Scale bar, 50 μm.

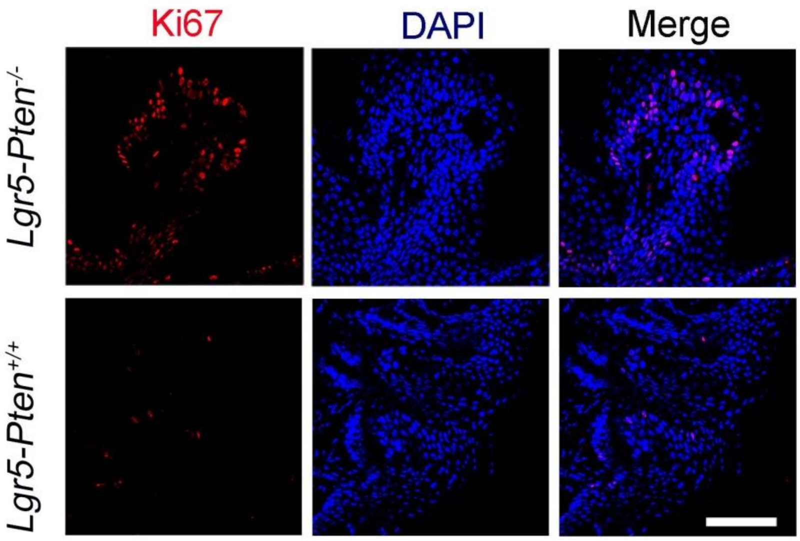

Figure S5. Ki67 expression in papillomata. Representative images of immune-fluorescence stain showing more Ki67 expressing cells in papilloma developed in *Lgr5-Pten*$^{-/-}$ mice compared to that in *Lgr5-Pten*$^{+/+}$ mice. Scale bar, 100 μm.

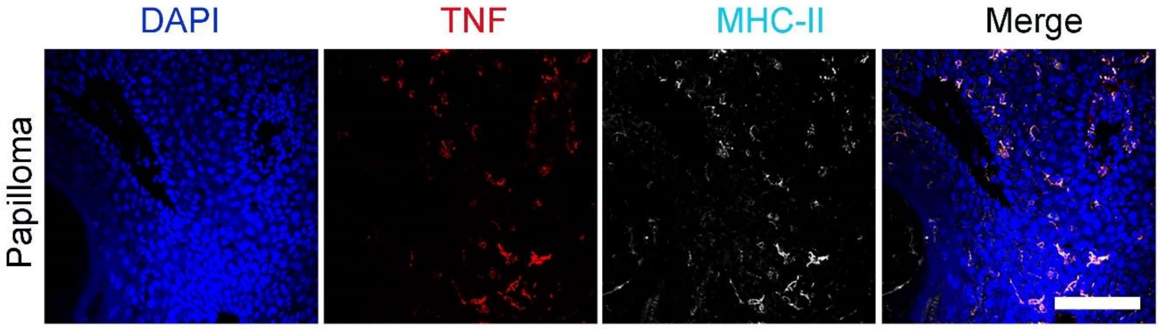

Figure S6. TNF expression in papilloma. Immunofluorescence analysis of papilloma tissue sections for the expression of TNF and MHC-II showed that TNF was largely present in MHC-II^{+} cells. Scale bar, 100 μm.
